# Dual-functional ROS-responsive hydrogel alleviates temporomandibular joint osteoarthritis by enhancing cartilage repair and mitigating inflammation

**DOI:** 10.1016/j.mtbio.2025.102103

**Published:** 2025-07-15

**Authors:** Yiwen Kuang, Bingqiang Hua, Xiaoping Ye, Yujin Zhao, Meng Yu, Xianwen Liu

**Affiliations:** a Department of Oral and Maxillofacial Surgery, Stomatological Hospital, School of Stomatology, Southern Medical University, Guangzhou, China; b NMPA Key Laboratory for Research and Evaluation of Drug Metabolism & Guangdong Provincial Key Laboratory of New Drug Screening & Guangdong-Hongkong-Macao Joint Laboratory for New Drug Screening, School of Pharmaceutical Sciences, Southern Medical University, Guangzhou, China

**Keywords:** Temporomandibular joint osteoarthritis, Injectable hydrogel, Reactive oxygen species, Cartilage repair, Regeneration

## Abstract

Temporomandibular joint osteoarthritis (TMJOA) is a degenerative disease characterized by variable degrees of inflammatory cartilage degeneration and subchondral bone erosion. Current clinical treatments mainly focus on symptomatic relief, such as pain management, and no disease-modifying osteoarthritis (OA) drugs (DMOADs) have been approved for widespread clinical use by regulatory bodies. Although fibroblast growth factor (FGF18) has shown great potential to be DMOADs in clinical trials for OA treatment by stimulating cartilage regeneration, its efficacy of inducing anabolic responses in articular cartilage is significantly limited by inflammatory conditions. In this study, we delivered FGF18 using an innovative hydrogel that underwent responsive degradation in the reactive oxygen species (ROS)-enriched TMJOA sites, continuously delivering the cartilage-repairing FGF18 and consuming ROS, further counteracting the limitation of inflammatory factors on cartilage repair ability of FGF18, thereby hindering the progression of TMJOA. This is the first attempt to hinder TMJOA progression with FGF18, and has demonstrated the dual function of hydrogel in reducing inflammation levels and promoting cartilage repair in TMJOA environment. This discovery addresses the limitations of short retention time of FGF18 in clinical applications, and also provides a new strategy for improving articular cartilage repair by overcoming the inflammatory environment.

## Highlights

• The injectable hydrogel degrades and releases FGF18 protein in response to ROS.
• FGF18 specifically targets inflammatory condylar chondrocytes for TMJOA therapy.
• The hydrogel inhibits inflammatory pathway to promote cartilage matrix regeneration.

## Graphical abstract

**Figure:**
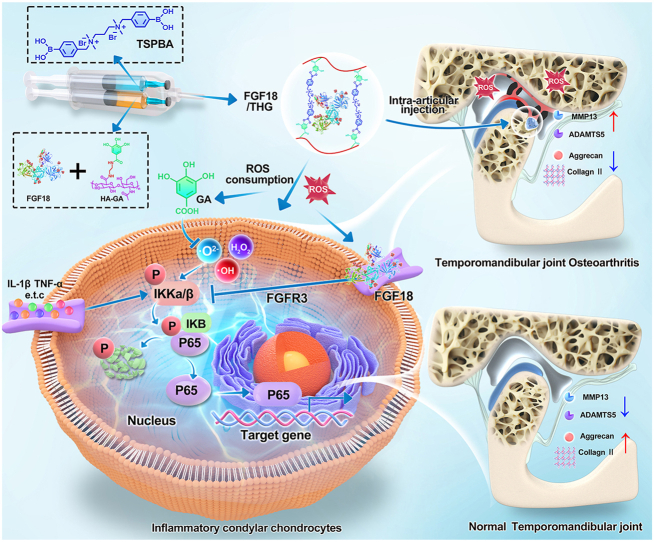

## 1 Introduction

Temporomandibular joint (TMJ) osteoarthritis (TMJOA) is a subtype of TMJ disorders (TMDs) characterized by varying degrees of inflammation, progressive cartilage degeneration, subchondral bone erosion, and chronic pain [1]. Approximately 40–75 % of adults experience symptoms of TMDs, including pain, restricted mandibular movement, and joint crepitus [2]. Traditional treatments for TMJOA primarily involve symptom-based non-surgical interventions, such as physical therapy to improve joint function, occlusal splints to adjust occlusion, nonsteroidal anti-inflammatory drugs to reduce inflammation and pain, and intra-articular injections of lubricants or corticosteroids to mitigate joint damage and pain [3,4]. However, current treatments cannot effectively reverse cartilage degradation or restore TMJ structure in progressive osteoarthritis (OA) [3]. This limitation arises from the unclear molecular mechanisms underlying cartilage degradation and extracellular matrix (ECM) loss in TMJOA, along with avascular cartilage's inherently low healing potential [5].

Oxidative stress, driven by excessive reactive oxygen species (ROS) production, and inflammation mediated by the nuclear factor (NF)-κB signaling pathway are associated with OA progression [6]. Specifically, increased ROS levels, decreased synthesis of antioxidant enzymes, and accumulation of lipid peroxidation products in the synovial fluid contribute to the oxidative stress implicated in OA pathophysiology [7,8]. NF-κB, a redox-sensitive transcription factor regulated by ROS levels, governs several processes, including inflammatory responses, cancer cell survival and invasion, and cytotoxicity [9,10]. Investigating these molecular mechanisms may provide new therapeutic approaches for TMJOA.

Disease-modifying osteoarthritis drugs (DMOADs), such as PG-116800 (a matrix metalloproteinase inhibitor), lorecivivint (SM04690, a Wnt pathway modulator), kartogenin, and sprifermin (recombinant human fibroblast growth factor 18, rhFGF18), have been explored for OA treatment [11,12]. Although their therapeutic efficacy has been demonstrated in animal studies, their safety and clinical efficacy remain unclear. As a result, none have received regulatory approval for widespread clinical use [12]. Among these, sprifermin activates FGFR3 on the surface of chondrocytes, modulating signaling pathways such as mitogen-activated protein kinase (MAPK) and phosphoinositide 3-kinase (PI3K), thereby regulating the runt-related transcription factor 2 (Runx2). This molecular mechanism drives chondrocyte proliferation and ECM synthesis. As a result, it decreases the expression of COL1, increases the COL2:COL1 ratio, and maintains chondrocyte phenotype, ultimately contributing to the repair of damaged cartilage [[13], [14], [15]]. Additionally, it inhibits the activity of cartilage-degrading enzymes, such as matrix metalloproteinase (MMP)13 and ADAM metallopeptidase with thrombospondin type 1 motif 5 (ADAMTS5), significantly reducing cartilage loss [16]. Sprifermin is currently in phase III clinical trials and has demonstrated a favorable safety profile with no reported serious local or systemic adverse events [17,18]. However, its clinical application is limited by its short half-life and low delivery efficiency, which necessitates repeated treatments [19,20]. Moreover, within inflammatory environments, sprifermin is significantly less capable of inducing anabolic responses in joint cartilage and stimulating type II collagen formation [19,21]. Consequently, sprifermin's ability to promote cartilage regeneration is more effective in OA patients with lower inflammation levels [21]. Therefore, strategies to mitigate the inflammatory environment could complement sprifermin-mediated joint cartilage repair and represent a promising therapeutic strategy for OA.

Hyaluronic acid (HA), a major component of the articular cartilage ECM, provides a structural framework for aggrecan binding, forming proteoglycan aggregates that confer resistance to compressive loads [22]. Due to its high biocompatibility and ease of modification with various functional groups, HA is frequently incorporated into hydrogel drug delivery systems, which improve drug retention and delivery profiles [[23], [24], [25]]. Indeed, HA-based hydrogels delivering therapeutic agents—from anti-inflammatory drugs to antisense nucleotides—have demonstrated efficacy in treating OA [[26], [27], [28]].

Excessive ROS, a hallmark of OA sites, exacerbates OA progression. The application of controllable biomaterials and personalized tissue engineering therapies offers a promising approach for the treatment of bone diseases [29]. Designing intra-articular drug delivery systems based on ROS-responsive materials, which can precisely regulate drug release in response to the oxidative stress state at OA lesion sites, is a promising strategy for OA treatment [30]. This approach not only enhances drug efficacy and reduces side effects but also effectively modulates local inflammatory responses, providing a novel solution for the treatment of OA. For instance, polymeric molecules with ROS-sensitive degradation properties, such as phenylboronic acid-modified levodopa and poly (lactic-co-glycolic acid), have been used to design OA drug delivery systems [[31], [32], [33]]. The novel engineered PON nanohybrids are designed for targeted delivery to lesion sites, effectively scavenging intracellular ROS [34]. Additionally, Furthermore, gas delivery materials facilitate the targeted delivery of hydrogen sulfide (H_2_S) and carbon monoxide (CO), further reducing intracellular ROS and reactive nitrogen species (RNS), thereby suppressing the excessive inflammation associated with OA [35,36]. These systems enable controlled, localized delivery of therapeutic agents in response to high ROS levels at the lesion site, reducing protein drug inactivation and off-target side effects. Small phenolic molecules such as gallic acid (GA) exhibit unique hydroxyl structures that facilitate oxidative free radical scavenging through hydrogen bonding or electron delocalization [37,38]. GA-based ROS-responsive drug delivery systems can effectively inhibit oxidative stress and reduce the expression of pro-inflammatory cytokines, including interleukin-1β (IL-1β) and tumor necrosis factor-α (TNF-α), eliciting significant anti-inflammatory effects [39,40]. These ROS-responsive platforms offer dual benefits: sustained drug delivery triggered by oxidative stress in the OA microenvironment, minimizing desensitization effects and drug toxicity, and effective neutralization of excessive ROS, enhancing cartilage repair by suppressing inflammation.

**Fig. 1:**
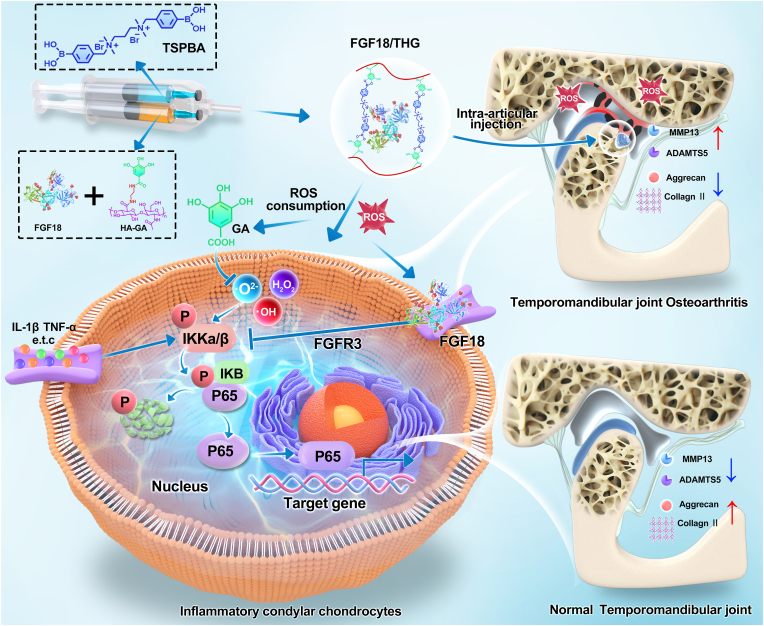
Schematic illustration of the mechanism by which ROS-sensitive THG hydrogel encapsulating FGF18 active protein (FGF18/THG) treats temporomandibular joint (TMJ) osteoarthritis (TMJOA). A ROS-responsive, biodegradable anti-inflammatory hydrogel (THG) is designed by combining HA–GA with N1-(4-boronobenzyl)-N3-(4-boronophenyl)-N1, N1, N3, N3-tetramethylpropane-1,3-diaminium (TSPBA). The hydrogel encapsulates FGF18 (FGF18/THG), which is injected into the TMJ cavity. Upon exposure to the reactive oxygen species (ROS)-enriched TMJOA microenvironment, the boronic ester groups in FGF18/THG degrade, leading to ROS depletion. Simultaneously, the released FGF18 and anti-inflammatory molecules (e.g., GA) further neutralize ROS and inhibit the abnormally activated NF-κB pathway in osteoarthritic condylar chondrocytes, upregulating cartilage-specific genes (*Col2*, *Acan*) and downregulating pro-inflammatory genes (e.g., *Adamts5, Mmp13*). This synergistic process promotes cartilage regeneration, reduces inflammation, and prevents extracellular matrix (ECM) degradation. Additionally, in milder cases of TMJOA, the therapeutic effects of FGF18 are further enhanced, slowing disease progression *in vivo*.

In this study, a novel injectable hydrogel composed of GA-modified HA and N1-(4-boronobenzyl)-N3-(4-boronophenyl)-N1, N1, N3, N3-tetramethylpropane-1,3-diaminium (TSPBA), referred to as THG, was developed to treat TMJOA by locally delivering the joint cartilage repair protein FGF18 (Fig. 1), a growth factor that promotes cartilage repair by stimulating the proliferation and differentiation of chondrocytes. The hydrogel matrix polymer is cross-linked via dynamic boronate ester bonds formed between the phenylboronic acid moieties in TSPBA and the o-diphenol groups of GA, with these bonds functioning as ROS-responsive linkers. Upon injection into the TMJ, the THG hydrogel degrades in response to elevated ROS levels in the TMJOA environment, enabling targeted local delivery of FGF18. This strategy shows potential to slow TMJOA progression caused by oxidative stress while enhancing cartilage regeneration via FGF18. Collectively, this research offers new avenues for improving joint disease treatment.

## 2 Materials and methods

### 2.1 Synthesis of FGF18/THG hydrogel

Sodium hyaluronate (MW: 100 kDa) was dissolved in deionized water, placed in a dialysis bag (MWCO: 3.5 KDa), dialyzed against a 0.01 M hydrochloric acid solution for two days to remove salts, and lyophilized to obtain sodium-free HA. Next, 10 g of HA was dissolved in 100 mL of dimethyl sulfoxide (DMSO), to which 15 g of N-hydroxysuccinimide (NHS) and 25 g of 1-ethyl-3-(3-dimethylaminopropyl) carbodiimide hydrochloride (EDC) was added to activate the carboxyl groups. After reacting overnight, 8 g of ethylenediamine was added, and the reaction continued for 6 h. The reaction mixture was placed in a dialysis bag (MWCO: 3.5 KDa) and dialyzed against deionized water for two days. The product was lyophilized to obtain HA–NH_2_.

Subsequently, 4 g of GA was dissolved in 50 mL of DMSO, to which 2.7 g of NHS and 4.5 g of EDC were added to activate the carboxyl groups for 6 h. In a separate reaction flask, 10 g of HA–NH_2_ was dissolved in 100 mL of DMSO. The activated GA solution was added dropwise to the HA–NH_2_ solution under a nitrogen atmosphere and allowed to react overnight. The reaction mixture was precipitated three times with ethanol and vacuum-dried to obtain a pale yellow HA–GA powder (Fig. 1A).

TSPBA was synthesized by mixing 4-(bromomethyl) phenylboronic acid (BMPBA) (1 g, 4.6 mmol) and N,N,N′,N'-tetramethyl-1,3-propanediamine (TMPDA) (0.2 g, 1.5 mmol) in 40 mL of dimethyl formamide (DMF) with constant stirring at 60 °C for 24 h. The clear solution was precipitated in 100 mL of tetrahydrofuran (THF) and filtrated. The white solid was washed with THF three times (20 mL each) and placed under vacuum overnight. Pure TSPBA (0.6 g, yield 70 %) was obtained as a white solid.

TSPBA and HA–GA were dissolved in phosphate-buffered saline (PBS) to a concentration of 3 % for TSPBA and 6 % for HA–GA. The resulting solutions and FGF18 (HY-P7124, MCE, New Jersey, NJ, USA) were stored at −20 °C for later use.

To prepare the FGF18/THG hydrogel, FGF18 was dissolved in the HA–GA solution. The TSPBA and HA–GA–FGF18 solutions were thoroughly mixed at a 1:1 vol ratio. After standing for 1 min, the mixture formed the FGF18/THG hydrogel. This ensured that FGF18 was uniformly distributed within the hydrogel and effectively encapsulated.

### 2.2 ROS-responsive local delivery capability of THG hydrogel

To evaluate the degradation behavior of the THG hydrogel and its localized release of FGF18 *in vitro*, 40 μL of the THG hydrogel was prepared by mixing 2 ng of FGF18, the HA-GA copolymer, and TSPBA, incubating at 37 °C for 1 min, and thoroughly washing. The hydrogel was then placed in 100 μL of PBS with or without 10 μM H_2_O_2_ and incubated at 37 °C. At predetermined time points (t) (0, 5, 10, 30, 60, 120 min), the buffer was collected, and the concentration of FGF18 released from the hydrogel was quantified using the FGF18 enzyme-linked immunosorbent assay (ELISA) Kit (ER2031, FineTest, Wuhan, China). After each collection, the remaining quantity of THG hydrogel (RQ) was recorded, and fresh PBS with or without H_2_O_2_ was replenished.

The remaining quantity of FGF18 (RQ) was calculated using Equation (1):

(1) $$RQ=q-\sum_{t=0}^{t}c_{t}V$$

where c_t_ represents the FGF18 concentration at time point *t*; *q* is the total initial quantity of FGF18 in the hydrogel; and *V* is the volume of fresh PBS added during each replenishment.

### 2.3 Isolation and culture of condylar chondrocytes

Mandibular condylar cartilage was extracted from 4-week-old female Sprague–Dawley (SD) rats, pooled, and digested with 0.25 % trypsin (Sigma, St. Louis, MO, USA) for 30 min. It was then digested for 8 h with type II collagenase (Sigma, V900892). The resulting chondrocytes were prepared as a single-cell suspension, counted, and seeded at a density of 1 × 10^5^ cells/cm^2^ in Dulbecco's modified eagle medium (DMEM/F12; Gibco, Waltham, MA, USA) with 10 % fetal bovine serum (FBS; ExCell Bio), 50 mg/mL streptomycin, and 50 units/mL of penicillin (Gibco). Cells were cultured to the second generation (P2) at 37 °C and 5 % CO_2_ for subsequent experiments.

### 2.4 Cytotoxicity assay

The cytotoxicity of the hydrogel on condylar chondrocytes was assessed after 24 and 48 h of treatment using the cell counting kit-8 (CCK-8) assay (APExBIO, K1081, Houston, TX, USA). Chondrocytes were seeded at a density of 2 × 10^4^ cells/well into 96-well plates and incubated overnight. Experimental groups were treated for 24 or 48 h with 0.5 μL of 4 ng/μL FGF18, 0.5 μL of THG hydrogel, or 0.5 μL of 4 ng/μL FGF18/THG hydrogel; the blank group was treated with an equivalent volume of DMEM. The assay was conducted in triplicate. Cell cytotoxicity was measured by determining the optical density at 450 nm using a microplate reader (BioTek Synergy H1, Agilent, Santa Clara, CA, USA).

### 2.5 *In vitro* oxidative stress analysis

P2 condylar chondrocytes, starved in DMEM with 1 % FBS, were divided into five groups. The IL-1β group was treated with 20 ng/mL IL-1β (211-11B, PeproTech, New Jersey, NJ, USA) for 24 h to induce inflammation. The FGF18, THG, and FGF18/THG groups were treated with 10 μL of 4 ng/μL FGF18, 10 μL of THG hydrogel, or 10 μL of 4 ng/μL FGF18/THG hydrogel, respectively, for 48 h. The control group received no treatment. On Day 3, total RNA and proteins were extracted for further analysis.

ROS levels were assessed by incubating cells with 10 μM dichloro-dihydro-fluorescein diacetate (DCFH-DA; S0033S, Beyotime, Shanghai, China) for 30 min and washed with PBS. Fluorescence was imaged at three random sites using a Leica DM IL LED microscope, and the average fluorescence intensity was quantified using Image software (NIH Image, National Institutes of Health, Bethesda, MD, USA).

### 2.6 RNA extraction and quantitative real-time PCR (qPCR)

Total RNA was extracted from rat condylar chondrocytes using TRIzol reagent (AG, AG21102, Hunan, China) according to the manufacturer's protocol. For cDNA synthesis, 1000 ng of total RNA was reverse transcribed using the Evo M-MLV RT Mix Kit with gDNA Clean for qPCR (AG, AG11728, Hunan, China). qPCR was conducted using a LightCycler 96 instrument (Roche, Switzerland) with the SYBR Green Premix Pro Taq HS qPCR Kit II (AG, AG11702, Hunan, China) to analyze the mRNA expressions of type II collagen (*Col2*)*,* aggrecan (*Acan*)*, Adamts5, Mmp13*, *Tnfa*, *Il6*, catalase (*Cat*), and superoxide dismutase 1 (*Sod1*) per the manufacturer's protocols. Relative mRNA levels were normalized to *Gapdh*, and differences in mRNA levels were calculated using the 2^−ΔΔCt^ method. The primers used for qPCR are listed in Table 1.

**Table 1:** Genes and primer sequences for real-time quantitative RT-PCR.

	Gene name	Forward primer sequence (5′–3′)	Reverse primer sequence (5′–3′)
**Primers for rat**	*Col2*	CACCGCTAACGTCCAGATGAC	GGAAGGCGTGAGGTCTTCTGT
	*Acan*	CCACTGGAGAGGACTGCGTAG	GGTCTGTGCAAGTGATTCGAG
	*Mmp13*	CTGCGGTTCACTTTGAGGAC	ACAGCATCTACTTTGTCGCC
	*Adamts5*	CACGACCCTCAAGAACTTTTGC	TCACATGAATGATGCCCACATAA
	*Tnfa*	GAGTGACAAGCCTGTAGCC	CTCCTGGTATGAGATAGCAA
	*Il6*	CACTTCACAAGTCGGAGGCT	AGCACACTAGGTTTGCCGAG
	*Cat*	AGAGGAAACGCCTGTGTGAG	TAGTCAGGGTGGACGTCAGT
	*Sod1*	TAACTGAAGGCGAGCATGGG	TCCCAATCACACCACAAGCC
	*Gapdh*	TGTTCTAGAGACAGCCGCATC	CACACCGACCTTCACCATCT

### 2.7 Western blotting

Chondrocytes were lysed in 1 × RIPA buffer (50 mM Tris (pH 7.4), 1 % Triton, 0.5 % Na-DCA, 0.1 % SDS, 150 mM NaCl, and 2 mM EDTA) supplemented with protease and phosphatase inhibitor cocktails (both from Beyotime). Protein concentrations were measured using the BCA kit (Beyotime). For each lane, 20 μg of total protein was loaded and separated on a 10 % Precast SDS–PAGE Gel (ACE, Nanjing, China) and transferred to polyvinylidene fluoride (PVDF) membranes (PR05505, Merck Millipore, Burlington, MA, USA). Membranes were blocked with 5 % bovine serum albumin (BSA; Solarbio, Beijing, China) for 1 h at room temperature and incubated overnight with primary antibodies specific for COL2 (1:3000, ab34712, Abcam) ACAN (1:4000, 13880-1-AP, Proteintech, Rosemont, IL, USA), ADAMTS5 (1:2000, ab41037, Abcam), MMP13 (1:4000, 18165-1-AP, Proteintech), phospho-NF-κB p65 (Ser536) (1:1000, #3033, CST, Danvers, MA, USA), NF-κB p65 (1:1000, #8242, CST), phospho-IKKα/β (Ser176/180) (1:1000, #2697, CST), IKKβ (1:1000, #8943, CST), and β-actin (1:5000, bs-0061R, Bioss, Peking, China). This was followed by incubation with appropriate horseradish peroxidase (HRP)-conjugated secondary antibodies (1:10,000, goat anti-Rabbit IgG, Servicebio, C1213). After washing, the blots were detected using enhanced chemiluminescence (ECL) (Merck Millipore) and visualized with a chemiluminescence system (Bio-Rad, Hercules, CA, USA), with image processing performed using Image Lab Software.

### 2.8 Establishment of a rat TMJOA model via partial discectomy

Female SD rats (2 months old) were obtained from the Guangdong Medical Experimental Animal Center and housed under controlled conditions (12:12 h light/dark cycle, 25 °C). All experimental procedures were approved by the Institutional Animal Care and Use Committee (IACUC) of Guangzhou Huateng Biopharmaceutical Technology Co. Ltd (IACUC NO: H7SW220937).

Thirty rats were divided into five groups (*n* = 6/group): (1) Sham surgery group (natural recovery, no treatment), (2) Control group (PDE + 50 μL PBS), (3) FGF18 group (PDE + 50 μL of 100 μg/mL FGF18), (4) THG hydrogel group (PDE + 50 μL THG hydrogel), and (5) FGF18/THG group (PDE + 50 μL of 100 μg/mL FGF18/THG hydrogel. TMJ partial discectomy (PDE) was performed as previously described, with the sham group undergoing a similar procedure without disk removal [41,42]. Four weeks post-surgery, intra-joint injections of FGF18, THG hydrogel, THG hydrogel loaded with FGF18, or PBS were administered. After eight weeks, the rats were euthanized using carbon dioxide, and the mandibular condylar cartilage structures were collected for subsequent histological and immunohistochemical (IHC) analysis.

### 2.9 Histological analysis and osteoarthritis research society international (OARSI) histopathological scoring

Osteoarthritic changes were assessed using a 0–4 grading system for joint cartilage degradation: Grade 0 (normal), Grade 1 (rough surface, minimal fibrillation or discoloration), Grade 2 (erosion into superficial/middle layers), Grade 3 (ulceration into deeper layers), and Grade 4 (cartilage depletion with subchondral bone exposure) [43]. TMJ condyles joints were dissected, and macroscopic changes were blindly evaluated by three observers.

Samples were fixed in 10 % formalin for 24 h, decalcified in 10 % EDTA (E1171, Solarbio) at 37 °C for eight weeks, and embedded in paraffin. Sections (4 μm thick) were stained with hematoxylin and eosin (H&E) and Safranin O/fast green (Solarbio, Cat#G1371). TMJ condyles were scored using a modified OARSI histological scoring system [41], as detailed in Table 2. Three independent observers blindly scored three random sections per sample.

**Table 2:** OARSI recommended histological scoring system.

Score	Parameter
	***Safranin O/fast green staining***
0	Uniform staining throughout articular cartilage
1	Loss of staining in superficial zone of hyaline cartilage <50 % the length of the condyle or plateau
2	Loss of staining in superficial zone of hyaline cartilage ≥50 % the length of the condyle or plateau
3	Loss of staining in the upper 2/3's of hyaline cartilage <50 % the length of the condyle or plateau
4	Loss of staining in the in the upper 2/3's hyaline cartilage ≥50 % the length of the condyle or plateau
5	Loss of staining in all the hyaline cartilage <50 % the length of the condyle or plateau
6	Loss of staining in all hyaline cartilage ≥50 % the length of the condyle or plateau
	***Structure***
0	Normal
1	Surface irregularities
2	Fissures in <50 % surface
3	Fissures in ≥50 % surface
4	Erosion 1/3 hyaline cartilage <50 % surface
5	Erosion 1/3 hyaline cartilage ≥50 % surface
6	Erosion 2/3 hyaline cartilage <50 % surface
7	Erosion 2/3 hyaline cartilage ≥50 % surface
8	Full depth erosion hyaline cartilage <50 % surface
9	Full depth erosion hyaline cartilage ≥50 % surface
10	Full depth erosion hyaline and calcified cartilage to the subchondral bone <50 % surface
11	Full depth erosion hyaline and calcified cartilage to the subchondral bone ≥50 % surface
	***Chondrocyte density***
0	No decrease in cells
1	Focal decrease in cells
2	Multifocal decrease in cells
3	Multifocal confluent decrease in cells
4	Diffuse decrease in cells
	***Cluster formation***
0	Normal
1	<4 clusters
2	4 ≤ clusters <8
3	≥8 clusters

### 2.10 Immunohistochemical analysis

Sections of TMJ condyle joints (4 μm) were deparaffinized, rehydrated, and incubated overnight at 4 °C with primary antibodies against COL2 (1:200, ab34712, Abcam), ACAN (1:400, 13880-1-AP, Proteintech), MMP13 (1:400, 18165-1-AP, Proteintech), ADAMTS5 (1:400, ab41037, Abcam), and phospho-IKKα/β (1:50, #2697, CST). A secondary antibody (goat anti-rabbit IgG; 1:200, C1213, Servicebio) and DAB (Goldbridge, Beijing, China) peroxidase color development kit were used for detection. The percentage of positive cells/area was quantified in three sequential sections from each condyle joint, with analysis performed in a blinded manner using ImageJ. Mean percentages were calculated for statistical analysis.

### 2.11 Immunofluorescence staining

The expression levels of TNF-α, IL-6, SOD1, CAT, and phospho-NF-κB p65 were analyzed using immunofluorescence staining. Chondrocytes were fixed with 4 % paraformaldehyde. Nonspecific staining was blocked using 5 % BSA. Subsequently, the cells were incubated overnight at 4 °C with primary antibodies specific for TNF-α (1:500, bs-10802R, Bioss), IL-6 (1:400, BA4339, Boster, Wuhan, China), SOD1 (1:500, bs-1079R, Bioss), CAT (1:500, bs-6874R, Boster), and phospho-NF-κB p65 (Ser536) (1:1000, #3033, CST). After washing, the cells were incubated with secondary antibodies (YF®488 goat anti-rabbit IgG, 1:500, Y6105, US Everbright, Suzhou, China) for 1 h. Nuclei were counterstained with 2-(4-amidinophenyl)-6-indolecarbamidine dihydrochloride (DAPI; Solarbio). Fluorescent images were captured using a fluorescence microscope (DMi8, Leica, Wetzlar, Germany). All procedures were conducted in the dark to preserve fluorescence signals.

### 2.12 Statistical analysis

Experimental data were processed using Prism 9 statistical software (GraphPad Inc., La Jolla, CA, USA). All values are presented as mean ± standard error of the mean (SEM). Statistically significant differences were assessed using an unpaired two-tailed Student's *t*-test for comparisons between two groups or one-way or two-way analysis of variance (ANOVA) followed by Tukey's multiple comparisons test for multiple group comparisons. Experiments were repeated at least three times, and a *P* value < 0.05 was considered statistically significant.

## 3 Results and discussion

### 3.1 Synthesis and characterization of THG hydrogel

**Fig. 2:**
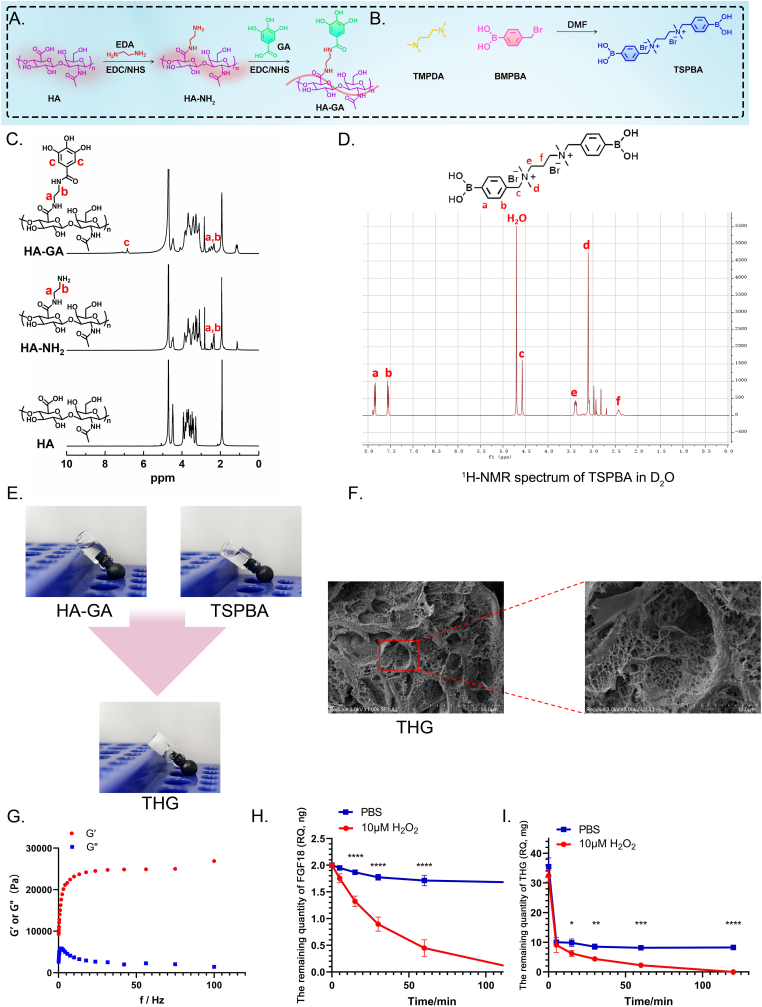
Characterization and properties of THG hydrogel. (A) Schematic of hyaluronic acid (HA)-gallic acid (GA) polymer synthesis, a precursor of THG hydrogel. (B) Schematic of N1-(4-boronobenzyl)-N3-(4-boronophenyl)-N1, N1, N3, N3-tetramethylpropane-1,3-diaminium (TSPBA) polymer synthesis, a precursor of THG hydrogel. (C) ^1^H NMR spectra of HA, HA–NH_2_, and HA–GA in D_2_O. (D) ^1^H NMR spectrum of TSPBA. (E) Schematic illustration of gel formation: HA–GA mixed with TSPBA at a 2:1 vol ratio forms an opaque white gel. (F) Scanning electron microscopy shows the formation of a porous network in THG hydrogel (scale bars: 50 μm and 10 μm). (G) Rheological evaluation of the storage (G′) and loss (G″) moduli of THG hydrogel at 37 °C. (H) Release profile of FGF18 from reactive oxygen species (ROS)-sensitive THG hydrogels immersed in 10 μM H_2_O_2_ or phosphate-buffered saline (PBS) solutions. (I) Degradation pattern of ROS-sensitive THG hydrogels immersed in 10 μM H_2_O_2_ or PBS solutions. Values are presented as the mean ± standard error of the mean (SEM) of three independent experiments. Significance was determined by Tukey's multiple comparison test following two-way analysis of variance (ANOVA). ∗*P* ≤ 0.05, ∗∗*P* ≤ 0.01, ∗∗∗*P* ≤ 0.001, ∗∗∗∗*P* < 0.0001.

The HA–GA copolymer was synthesized through an NHS/EDC coupling reaction using sodium HA and GA (Fig. 2A). Subsequently, a nucleophilic substitution reaction between the nitrogen atoms in the nucleophilic reagent TMPDA and the bromomethyl groups in BMPBA yielded the quaternary ammonium salt product TSPBA (Fig. 2B).

Proton nuclear magnetic resonance (^1^H NMR) spectroscopy in D_2_O solvent verified the successful synthesis of the intermediate product HA–NH_2_ and the final product HA–GA (Fig. 2C). The characteristic peaks a and b of HA–NH_2_ appear between 2 and 3 ppm, exhibiting doublet or quartet signals, which correspond to the methylene (-CH2-) protons of HA–NH_2_ [44]. In contrast, the HA–GA copolymer exhibited an additional c peak in the 6–7 ppm range, representing the hydrogen atoms on the aromatic ring (phenyl group) of the GA precursor. These results strongly confirm the successful synthesis of the HA–GA copolymer and provide critical structural information.

The TSPBA was characterized using ^1^H NMR spectroscopy, and the results were consistent with the expected chemical structure and properties (Fig. 2D). Specifically, peaks a and b correspond to the aromatic ring protons in TSPBA, peak c represents the methylene protons (–CH2–) of the bridging group, and peak d is attributed to the protons connected to the amine group (–NH). These findings confirm the successful synthesis of the ROS-responsive linker [45].

At 37 °C, aqueous solutions of the HA–GA copolymer and TSPBA were clear and transparent. When mixed and extruded at a 2:1 vol ratio using a dual-channel syringe, these solutions underwent a controllable crosslinking process for approximately 1 min, resulting in the formation of a white gel, i.e., THG (Fig. 2E). The gel remained stable and did not flow under gravity. This phenomenon was attributed to the efficient formation of boronate ester bonds between the phenylboronic acid groups of TSPBA and the vicinal dihydroxyl groups of GA during the injection process, leading to rapid crosslinking and gelation, thus demonstrating the excellent *in situ* gelation properties of THG.

Scanning electron microscopy (SEM, SU8100, HITACHI, Japan) revealed the internal structure of the THG hydrogel as a distinct porous network (Fig. 2F). The micropores of THG, approximately 10 μm in diameter, were significantly larger the FGF18 protein (diameter: ∼2.7–3 nm). This unique microporous structure enhanced the loading capacity for therapeutic FGF18 and facilitated its rapid delivery, making THG an ideal platform for active drug delivery.

A frequency sweep test was conducted to evaluate the dynamic mechanical properties of the fully crosslinked THG hydrogel. A fixed strain amplitude of 1 % was applied while monitoring changes in the storage modulus (G′) and loss modulus (G″) over a frequency range of 0.1–100 Hz (Fig. 2G). The results indicated that at 37 °C, the G′ of the THG hydrogel was significantly higher than the G″, demonstrating that its physical properties were more akin to those of a flexible hydrogel than a viscous liquid.

### 3.2 ROS-responsive degradation and drug delivery capability

ROS are key contributors to the progression of OA [39]. They induce oxidative stress, disrupt cartilage homeostasis, and promote chondrocyte apoptosis and DNA damage. ROS also activate pro-inflammatory cytokines, such as MMPs and IL-1β [40], which exacerbate ROS production, accelerate cartilage degradation, and promote OA progression. TSPBA is a boronic acid-based crosslinker that not only reacts with the hydroxyl groups of vicinal diols in the HA-GA polymer to form boronate ester bonds, leading to rapid gel formation, but also exhibits a crucial feature: as an ROS-responsive linker, the aromatic boronate ester primarily coordinates with the boron atom through H_2_O_2_, resulting in the oxidation of the B–C bond to form borate salts [46]. These can rapidly hydrolyze in water into boric acid/esters and aromatic phenols (which may further transform into quinones), simultaneously facilitating structural breakdown and the localized release of encapsulated FGF18 [47]. The gel degradation and drug delivery behavior were compared in PBS (pH 7.4) and the lesion environment. ELISAs were employed to measure FGF18 release at different time points, along with monitoring hydrogel mass changes (Fig. 2H and I).

Under physiological conditions, the THG hydrogel exhibited a degradation rate of approximately 28 % within 2 h, with FGF18 release limited to 18 %. In contrast, in the ROS environment, the ROS-responsive boronate ester crosslinked structure of FGF18/THG resulted in rapid degradation, with cumulative FGF18 release approaching 100 % within 2 h. These findings suggest that FGF18/THG effectively responds to oxidant environments, releasing therapeutic agents triggered by excessive ROS and scavenging ROS, thereby mitigating tissue damage caused by the oxidant microenvironment.

However, we also observed a significant initial mass loss of the THG hydrogel in PBS without the addition of H_2_O_2_. This phenomenon is likely attributable to a mismatch between the crosslinking kinetics and the measurement time window, leading to incomplete bonding of some TSPBA, which subsequently dissolved rapidly in PBS. As a result, a portion of the HA-GA remained un-crosslinked, exhibiting an initial degradation rate similar to that observed under ROS conditions. However, over time, the THG hydrogel gradually stabilized. At later time points (10, 30, 60, and 120 min), the THG hydrogel demonstrated distinctly different behavior under physiological conditions compared to the ROS environment.

### 3.3 *In vitro* evaluation of the THG hydrogel cytotoxicity in condylar chondrocytes and its inhibitory effect on inflammation-related ROS

**Fig. 3:**
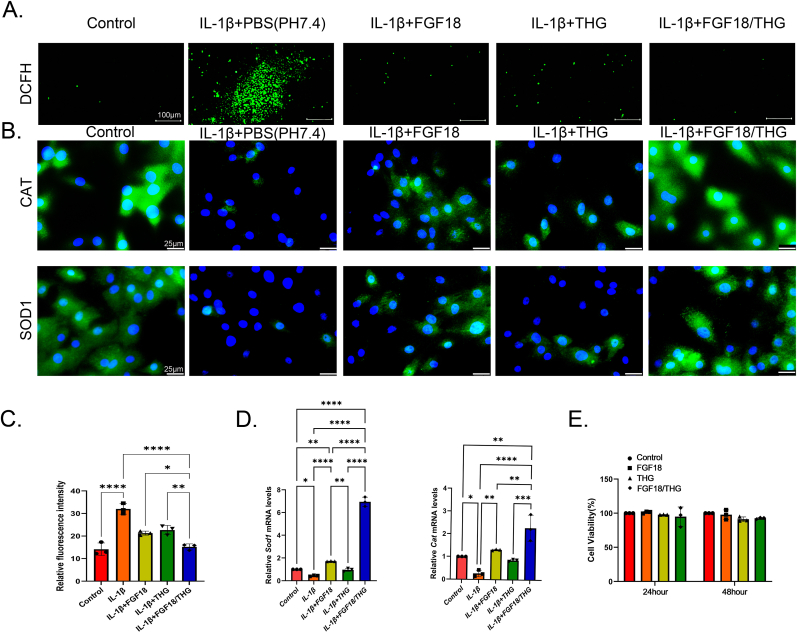
*In vitro* anti-inflammatory and cytotoxicity evaluation of FGF18/THG. (A) Representative fluorescence images of the reactive oxygen species (ROS) levels in condylar chondrocytes co-cultured with FGF18, THG, and FGF18/THG using ROS fluorescence staining. (B) Immunofluorescence staining of catalase (CAT) and superoxide dismutase 1 (SOD1) expression in IL-1β-induced condylar chondrocytes treated with FGF18/THG. (C) Quantitative evaluation of ROS levels in condylar chondrocytes using ROS fluorescence staining. (D) mRNA expression of *Cat* and *Sod1* in IL-1β-induced condylar chondrocytes treated with FGF18/THG. (E) CCK-8 assay results showing the cytotoxicity of FGF18/THG on condylar chondrocytes based on cell proliferation. Values are presented as the mean ± standard error of the mean (SEM) of three independent experiments. Significance was determined by one-way analysis of variance (ANOVA): ∗*P* ≤ 0.05, ∗∗*P* ≤ 0.01, ∗∗∗*P* ≤ 0.001, ∗∗∗∗*P* < 0.0001.

Elevated levels of IL-1β in osteoarthritic cartilage promote the production of matrix-degrading enzymes while suppressing the synthesis of ECM proteins by chondrocytes [48]. Consequently, IL-1β is a key cytokine extensively studied in the progression of OA [49]. In this study, DCFH-DA was employed as a probe to detect ROS levels in IL-1β-induced inflammatory OA chondrocytes co-cultured with the THG hydrogel, using high-resolution fluorescence microscopy. The relative mean fluorescence intensity was quantified (Fig. 3A–C). Additionally, qRT-PCR and immunofluorescence staining were used to evaluate the activity of antioxidant factors in IL-1β-induced chondrocytes under different treatments, with particular focus on SOD1 and CAT.

SOD1 catalyzes the conversion of superoxide anions (O_2_·) into less harmful hydrogen peroxide (H_2_O_2_), which is subsequently reduced to water by CAT [50]. This compensatory antioxidant mechanism provides cellular protection and slows OA progression [51]. The results showed that, compared to the positive IL-1β group (IL-1β treatment alone), primary mandibular condylar chondrocytes from SD rats exhibited significantly reduced ROS levels and increased expression of CAT and SOD1 following treatment with either FGF18 or the THG hydrogel (Fig. 3B–D). Notably, ROS levels in the FGF18/THG group were significantly lower than in the FGF18 or THG hydrogel groups alone, approaching levels observed in the control group. Moreover, CAT and SOD1 expression levels were markedly higher in the FGF18/THG group than in any other treatment group.

The boronate ester bonds in the THG hydrogel structure rapidly respond to ROS, undergoing cleavage that significantly reduces ROS levels in oxidative environments and induces hydrogel disintegration. Additionally, the monohydroxy and phenolic radicals in the hydrogel material further scavenge ROS and related radicals produced by inflammatory cells, effectively mitigating tissue damage caused by oxidative stress. As the hydrogel gradually degrades, the encapsulated protein drug FGF18 is slowly released, exerting its effects on inflammatory condylar chondrocytes. Upon selective activation of FGFR3 by its homologous ligand FGF18 [52], a dual regulatory effect is exerted: On the one hand, it inhibits excessive chondrocyte proliferation, promotes the differentiation of mesenchymal cells, and maintains the anabolic balance of articular cartilage [53]; on the other hand, FGF18 enhances the activity and expression of the downstream effector, the tyrosine kinase FYN, which in turn negatively regulates the NADPH oxidase 4 (NOX4) and suppresses the aberrant generation of ROS [54].

Subsequently, the impact of FGF18/THG on condylar chondrocyte cytotoxicity was assessed using the CCK-8 assay. No significant differences were observed in the viability of primary condylar chondrocytes from SD rats co-cultured with FGF18 or THG hydrogel for 1 or 2 days compared to the untreated control group (Fig. 3E). These findings confirm the excellent biocompatibility of the THG hydrogel with condylar chondrocytes, highlighting its potential for biomedical applications.

### 3.4 FGF18/THG protects condylar chondrocytes from IL-1β-induced inflammatory conditions

The cartilage-specific ECM secreted by chondrocytes is critical for cartilage regeneration and homeostasis, with COL2 and ACAN serving as key components and markers of cartilage ECM [55]. MMPs and ADAMTS5 are central to the progression of OA and the degradation of cartilage ECM [56]. Thus, to evaluate the impact of FGF18/THG on ECM synthesis in OA condylar chondrocytes, an IL-1β-induced OA condylar chondrocyte model was established, and chondrocytes were co-cultured with FGF18, THG hydrogel, or FGF18/THG, respectively.

**Fig. 4:**
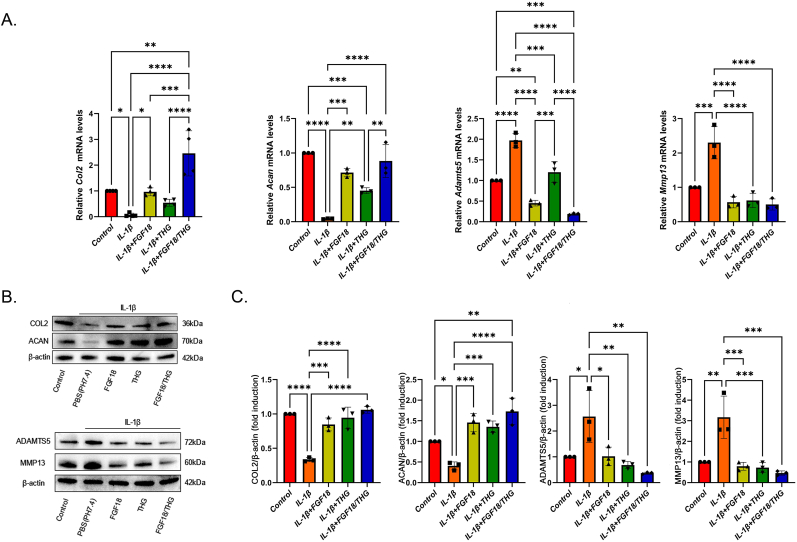
Effect of FGF18/THG on cartilage matrix synthesis and degradation in IL-1β-induced inflamed condylar chondrocytes. (A) RT-qPCR analysis of relative mRNA expression levels of *Col2*, *Acan*, *Adamts5*, and *Mmp13* in IL-1β-induced condylar chondrocytes co-cultured with FGF18/THG. (B) Western blot detection of COL2, ACAN, ADAMTS5, and MMP13 proteins in IL-1β-induced inflamed condylar chondrocytes co-cultured with FGF18/THG. (C) Semi-quantitative analysis of the immunoblotting results. Values are presented as the mean ± standard error of the mean (SEM) of three independent experiments. Significance was determined by one-way analysis of variance (ANOVA): ∗*P* ≤ 0.05, ∗∗*P* ≤ 0.01, ∗∗∗*P* ≤ 0.001, ∗∗∗∗*P* < 0.0001. The cells were primary condylar chondrocytes isolated from Sprague–Dawley (SD) rats.

RT-qPCR analysis showed significantly higher expression levels of cartilage-specific matrix genes *Col2* and *Acan* in OA condylar chondrocytes treated with FGF18/THG compared with the IL-1β group. Additionally, the expression of ECM degradation-related genes *Adamts5* and *Mmp13* was markedly reduced (Fig. 4A). Western blot analysis corroborated these findings, indicating that FGF18/THG effectively reduces proteoglycan loss in OA chondrocytes, significantly enhances ECM synthesis and quality, and inhibits the expression of ECM degradation-related proteins (Fig. 4B and C). Furthermore, FGF18 and the THG hydrogel exhibited anti-inflammatory effects compared with the IL-1β group.

The THG hydrogel, through ROS-responsive degradation, reduced ROS levels in the surrounding environment and facilitated the localized delivery of the small molecule GA, which scavenges free radicals. This process exhibited anti-inflammatory activity, diminished IL-1β-induced pro-inflammatory effects, and suppressed ECM degradation-related protein expression. FGF18 is a high-affinity ligand for FGFR3, which, upon binding to FGFR3 on the cell membrane, activates the MAPK, ERK1/2, and Hedgehog signaling pathways to regulate chondrocyte proliferation [57,58]. Additionally, by activating the ERK1/2 signaling pathway, FGF18 promotes the expression of type II collagen (COL2) and Sox-9, while inhibiting the expression of MMP-13 and ADAMT-5. It also enhances the transcription of RUNX2, thereby stimulating the synthesis of extracellular matrix in cartilage [59]. Thus, FGF18/THG effectively reversed the catabolic imbalance induced by inflammation.

### 3.5 Pharmacodynamics evaluation of FGF18/THG in restoring condylar cartilage in a TMJOA rat model

**Fig. 5:**
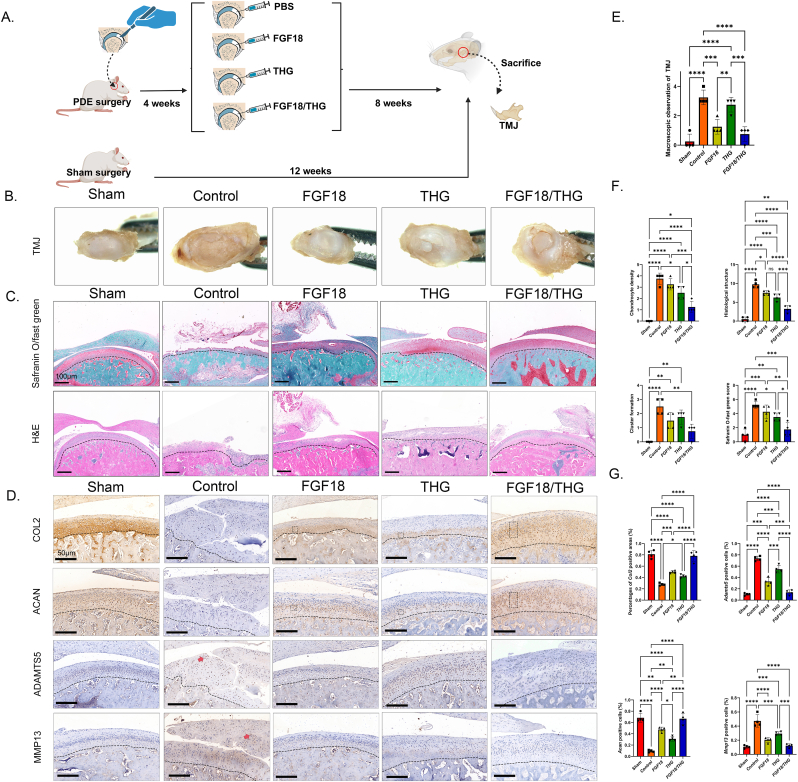
Macroscopic evaluation and histopathological assessment of the temporomandibular joint (TMJ) in SD rats with experimental TMJ osteoarthritis (TMJOA) after two weeks of intra-joint injection treatment with FGF18/THG. (A) Schematic diagram of the SD rat TMJOA experimental model, indicating the time points for partial discectomy, intra-joint injection of FGF18, THG, or FGF18/THG, and sacrifice. (B) Representative macroscopic images of the TMJ condyles in sham-operated and PDE-operated rats after eight weeks of treatment with FGF18/HA–GA or control. (C) Representative histological images stained with H&E and Safranin O/fast green showing histological changes in cartilage sections from different groups of SD rats eight weeks post-PDE surgery. (D) Representative immunohistochemical images of COL2, ACAN, ADAMTS5, and MMP13 staining in the TMJ condyles of rats two weeks post-sham surgery or PDE surgery, treated with FGF18/THG or control. The dashed box highlights the repair area, and arrows indicate the inflammatory regions. (E) Macroscopic semi-quantitative scoring of the TMJ condylar cartilage in SD rats. (F) Semi-quantitative scoring of Safranin O/fast green staining in the TMJ condylar cartilage of different groups of SD rats. (G) Semi-quantitative analysis of COL2, ACAN, ADAMTS5, and MMP13 immunohistochemical staining in TMJ cartilage of different groups of SD rats. Values are presented as the mean ± standard error of the mean (SEM) of four independent experiments. Significance was determined by one-way analysis of variance (ANOVA): ∗*P* ≤ 0.05, ∗∗*P* ≤ 0.01, ∗∗∗*P* ≤ 0.001, ∗∗∗∗*P* < 0.0001.

Using a PDE-based model, the therapeutic effects of FGF18/THG in mitigating progressive cartilage loss and damage in TMJOA were investigated (Fig. 5A). To comprehensively evaluate the individual contributions of FGF18 and THG hydrogel, five treatment groups were compared: (1) Sham surgery group (natural recovery, no treatment), (2) Control group (PDE + PBS treatment), (3) FGF18 group (PDE + FGF18 treatment only), (4) THG hydrogel group (PDE + THG hydrogel treatment only), and (5) FGF18/THG group (PDE + FGF18/THG hydrogel treatment). By comparing the control group with the FGF18, THG hydrogel, and FGF18/THG groups, the distinct and combined effects of FGF18 and THG were analyzed.

Gross observations (Fig. 5B) after eight weeks revealed pronounced OA characteristics in the control group, including widespread and severe cartilage surface irregularities, with subchondral bone visibly exposed in some cases. These findings confirm the successful replication of typical OA features in the TMJOA model. In contrast, experimental groups exhibited varying degrees of morphological improvement. Rats treated with FGF18 or the THG hydrogel showed minor surface irregularities, demonstrating the cartilage repair effects of FGF18 and the lubricating, ROS-scavenging, and anti-inflammatory properties of the THG hydrogel. Notably, the FGF18/THG group displayed nearly smooth cartilage surfaces with minimal visible damage, resembling the natural group in structural integrity. Macroscopic semi-quantitative scores (Fig. 5E) supported these findings, with the FGF18/THG group scoring lower than the FGF18 and THG groups, highlighting the combined therapeutic advantages of FGF18 and the THG hydrogel in cartilage repair.

H&E staining and Safranin O/fast green staining were performed to assess the structure, proteoglycan content, and collagen deposition in the condylar cartilage. Results (Fig. 5C–F) indicated severe damage in the control group, including rough cartilage surfaces, vertical fissures, fibrosis, erosion, and reduced chondrocyte density. In contrast, the sham surgery and FGF18/THG groups retained cartilage structural integrity at week 8, with smooth cartilage surfaces, increased chondrocyte density, reduced clustering, and uniformly distributed proteoglycans in the ECM (as shown by Safranin O staining). The modified OARSI semi-quantitative scoring system demonstrated that the FGF18/THG group had the lowest score, comparable to the sham surgery group, underscoring its therapeutic effect in mitigating TMJOA-induced cartilage damage. Based on the red-stained area and intensity in Safranin O/fast green staining, the FGF18/THG group exhibited the greatest potential in combating OA-related cartilage matrix degradation.

IHC analysis of cartilage ECM, inflammation, and matrix degradation-related proteins (Fig. 5D–G) showed that the FGF18/THG group significantly increased the expression of ECM proteins such as COL2 and ACAN while reducing the expression of inflammatory and degradation-related proteins ADAMTS5 and MMP13. Conversely, the control group exhibited elevated ADAMTS5 and MMP13 expression, further confirming the reparative potential of FGF18/THG in TMJOA by promoting ECM protein synthesis and inhibiting inflammation and matrix degradation. These findings align with previous *in vitro* results.

These results indicate that FGF18/THG injection effectively eliminates ROS within the TMJOA environment through ROS-responsive degradation of the THG hydrogel while delivering FGF18 and GA. The synergistic actions of GA and FGF18 regulate chondrocyte degradation and synthesis activities, facilitating their transition to normal chondrocytes and enhancing cartilage repair. Furthermore, FGF18 demonstrates greater efficacy in low-ROS environments, improving therapeutic outcomes. This process establishes a feedback mechanism that reduces ROS levels, halting TMJOA progression.

Additionally, intra-articular injection of FGF18 promoted ECM protein synthesis, increased cartilage thickness, and inhibited cartilage matrix-degrading enzyme activity, significantly slowing cartilage degradation [20,60]. A single THG hydrogel injection also decelerated TMJOA progression, likely due to its sustained localized delivery of anti-inflammatory molecules within the inflamed joint cavity. Moreover, the THG hydrogel forms a protective layer, reducing friction and wear during joint movement, enhancing cell signaling, promoting cell adhesion, and modulating inflammation. These combined effects effectively alleviate pain and inflammation [61].

### 3.6 FGF18/THG protects cartilage from inflammatory damage by inhibiting NF-κB pathway activation

The NF-κB is a family of transcription factors crucial for regulating inflammation, differentiation, proliferation, and survival in mammalian cells [62]. Various pro-inflammatory cytokines, such as IL-1β and TNFα, activate IκB kinases (IKKs), leading to IκB degradation through phosphorylation [63]. This allows the NF-κB complex to translocate to the nucleus, where it regulates the transcription of downstream target genes and promotes the secretion of cartilage matrix-degrading enzymes, including MMP13 and ADAMTS5, ultimately resulting in joint cartilage degradation [64,65]. Consequently, the NF-κB signaling pathway is recognized as a therapeutic target for OA [66]. Glucocorticoids, for example, are widely used to treat inflammatory diseases [67]. Glucocorticoids upregulate the expression of NF-κB inhibitory proteins, such as IκB, and prevent NF-κB from binding to DNA, thereby inhibiting transcription [67]. In addition, receptor antagonists, such as TNF receptor inhibitors, IL-1β receptor antagonists, IKK inhibitors, and NF-κB nuclear translocation inhibitors, are emerging as new therapeutic approaches for OA [[68], [69], [70]].

**Fig. 6:**
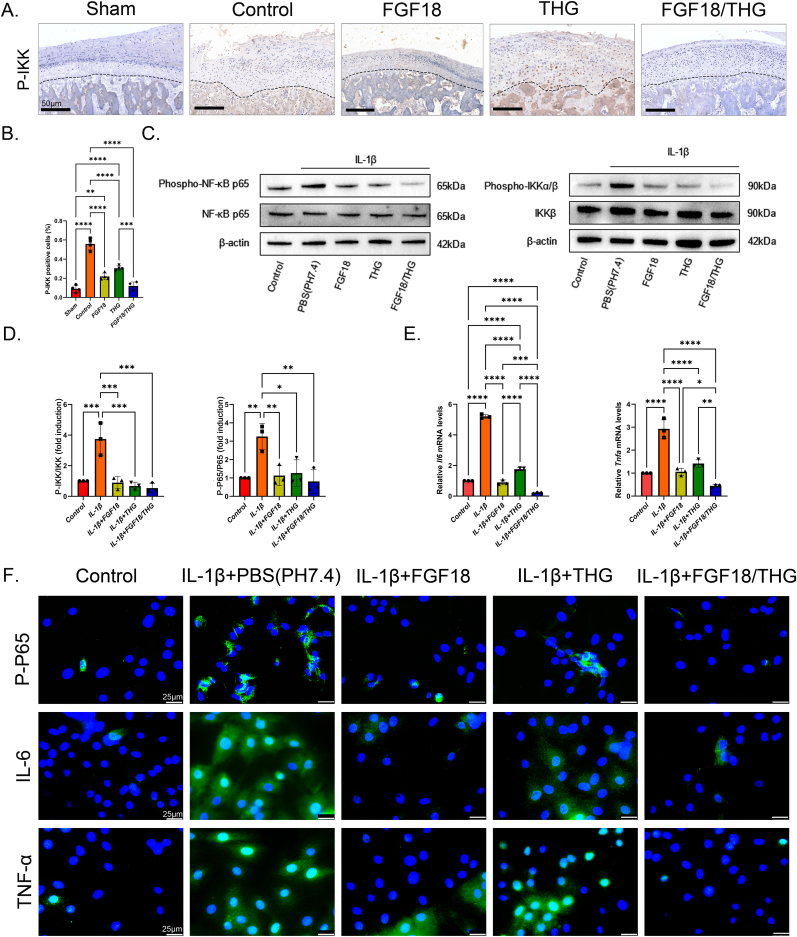
FGF18/HA–GA treats temporomandibular joint osteoarthritis (TMJOA) by inhibiting the NF-κB pathway. (A) Representative p-IKK immunohistochemical staining images of temporomandibular joint (TMJ) condyles from SD rats treated with FGF18/HA–GA or control, eight weeks post-sham surgery and partial discectomy (PDE) surgery. (B) Semi-quantitative analysis of p-IKK immunohistochemical staining in TMJ cartilage of different groups of SD rats. (C) Western blot analysis of p-IKK and p-p65 protein expression levels in IL-1β-induced inflamed condylar chondrocytes co-cultured with FGF18/THG. (D) Semi-quantitative analysis of the immunoblotting results for each group. Values are presented as the mean ± standard error of the mean (SEM) of three independent experiments. (E) Relative mRNA expression levels of *Tnfa* and *Il6* in IL-1β-induced condylar chondrocytes co-cultured with FGF18/THG. (F) Protein expression of p-p65, TNF-α, and IL-6 in IL-1β-induced condylar chondrocytes co-cultured with FGF18/THG. Significance was determined by one-way analysis of variance (ANOVA): ∗*P* ≤ 0.05, ∗∗*P* ≤ 0.01, ∗∗∗*P* ≤ 0.001, ∗∗∗∗*P* < 0.0001.

We hypothesize that FGF18/THG downregulates the NF-κB signaling pathway, promoting ECM regulation while inhibiting inflammation and matrix degradation. The proposed molecular mechanism is depicted in Fig. 1. To test this hypothesis, immunohistochemical staining was performed to evaluate p-IKK, a key NF-κB signaling protein, in a TMJOA rat model. IKK is part of the upstream cascade of NF-κB signaling, where phosphorylation of IκBα at Ser32/Ser36 triggers its degradation via the ubiquitin-proteasome pathway. In TMJOA, this modification facilitates the nuclear translocation of the p65/p50 heterodimer, activating the transcription of matrix metalloproteinases (such as MMP13) and aggrecanases (such as ADAMTS-5), leading to irreversible degradation of cartilage matrix components like type II collagen and proteoglycans [67]. The FGF18/THG group was compared with the PBS (control) group. The results (Fig. 6A and B) revealed a high number of p-IKK-positive cells in the condylar cartilage layer of the control group, indicating persistent NF-κB pathway activation, consistent with TMJOA pathology. In contrast, the FGF18/THG group exhibited significantly fewer p-IKK-positive cells, similar to the sham-operated group. Furthermore, *in vitro* experiments corroborated the immunohistochemical findings (Fig. 6C and D). Specifically, IL-1β treatment resulted in significantly elevated levels of p-IKK and p-NF-κB-p65, while both FGF18 and THG hydrogel treatments effectively inhibited their upregulation. In the FGF18/THG treatment group, the expression levels of p-IKK and p-NF-κB-p65 were significantly lower than in the IL-1β group and were comparable to the control group. Additionally, cell immunofluorescence staining further validated the impact of FGF18/THG treatment on the expression of downstream pro-inflammatory factors in IL-1β-induced OA condylar chondrocytes (Fig. 6E and F). The results showed that FGF18/THG treatment inhibited the nuclear translocation of p-p65 in OA chondrocytes, thereby reducing the expression of downstream pro-inflammatory cytokines, IL-6 and TNFα.

These findings support the hypothesis that intra-articular injection of FGF18/THG improves the ROS-enriched microenvironment of TMJOA. Locally delivered FGF18, along with anti-inflammatory molecules such as GA, inhibits the phosphorylation of key proteins (such as IKK) in the aberrantly activated NF-κB pathway in OA condylar chondrocytes, preventing the phosphorylation of p65 from translocating into the nucleus. This process enhances the expression of cartilage-specific genes while downregulating pro-inflammatory genes. This synergistic action promotes cartilage regeneration, reduces inflammation, prevents ECM degradation, and alleviates TMJOA progression *in vivo*.

This study has several limitations that should be addressed in future research. First, the subchondral bone, which plays a crucial role in osteoarthritis progression, was not analyzed. Including its analysis would provide a more comprehensive understanding of the FGF18/THG hydrogel effects on the entire joint. Second, the study primarily focused on short-term effects, and the long-term efficacy of the FGF18/THG hydrogel remains unclear. Further investigation is needed to evaluate whether the FGF18/THG hydrogel exerts a sustained therapeutic impact over time. Finally, the *in vitro* conditions used in the study may not fully replicate the complexities of the *in vivo* environment. Additional research is required to assess the hydrogel performance in more clinically relevant settings, taking into account factors such as joint mechanics, immune response, and systemic influences that could affect its therapeutic potential.

## 4 Conclusion

In this study, an innovative therapeutic strategy is proposed to halt TMJOA progression, involving the use of HA–GA and TSPBA to synthesize a THG hydrogel as a drug carrier, with FGF18 as the active agent. The FGF18/THG system leverages unique boronate ester bonds to continuously scavenge ROS within the TMJ, reducing inflammation in condylar chondrocytes. Moreover, the THG hydrogel enables the localized delivery of FGF18 and the small molecule GA, which synergistically inhibit the NF-κB signaling pathway in inflammatory condylar chondrocytes. This dual mechanism reverses ECM degradation, promotes cartilage matrix synthesis, alleviates the inflammatory environment, and effectively halts TMJOA progression. The findings offer a novel therapeutic approach for TMJOA and provide a potential reference for treating other forms of OA. Future research should examine the long-term effects of this treatment strategy and its applicability across diverse patient populations, advancing OA therapy.

## CRediT authorship contribution statement

**Yiwen Kuang:** Writing – original draft, Formal analysis, Data curation. **Bingqiang Hua:** Formal analysis, Data curation. **Xiaoping Ye:** Validation, Formal analysis. **Yujin Zhao:** Validation. **Meng Yu:** Writing – review & editing, Resources, Conceptualization. **Xianwen Liu:** Writing – review & editing, Data curation, Conceptualization.

## Declaration of competing interest

The authors declare that they have no known competing financial interests or personal relationships that could have appeared to influence the work reported in this paper.

## Data Availability

Data will be made available on request.
